# Supplementary material for: Improvement of renal function after transcatheter aortic valve replacement in patients with chronic kidney disease
Source: PLoS One. 2021 May 13;16(5):e0251066. doi: 10.1371/journal.pone.0251066 (PMC8118303; doi:10.1371/journal.pone.0251066)
Supplement: S1 Table — (DOCX) [file pone.0251066.s001.docx]

**S1 Table. Baseline and procedural characteristics of patients with loss of follow-up versus patients with complete 1-year follow-up.**

|  | **Loss of Follow-up Group** | **Complete 1-Year Follow-up Group** | **Overall** |  |
| --- | --- | --- | --- | --- |
|  | **(n = 157)** | **(n = 420)** | **(n = 577)** | **P value** |
| **Clinical data** |  |  |  |  |
| Age, years | 81.8 ± 5.7 | 81.9 ± 7.2 | 81.3 ± 6.8 | 0.86 |
| Male sex | 92 (58.6) | 232 (55.2) | 324 (56.2) | 0.47 |
| NYHA class III/IV | 127 (80.9) | 354 (84.3) | 481 (83.4) | 0.33 |
| Diabetes | 44 (28.0) | 139 ( 33.1) | 183 (31.7) | 0.24 |
| Hypertension | 126 (80.3) | 304 (72.4) | 430 (74.5) | 0.053 |
| COPD | 29 (18.5) | 82 (19.5) | 111 (19.2) | 0.77 |
| Pulmonary hypertension | 37 (23.6) | 101 (24.0) | 138 (23.9) | 0.90 |
| CAD | 103 (65.6) | 248 (59.0) | 351 (60.8) | 0.15 |
| Peripheral vascular disease | 31 (19.7) | 65 (15.5) | 96 (16.6) | 0.22 |
| Previous CABG | 36 (22.9) | 84 (20.0) | 120 (20.8) | 0.44 |
| STS score, % | 10.1 ± 7.4 | 10.8 ± 8.1 | 10.6 ± 7.9 | 0.29 |
| eGFR, mL/min/1.73m^2^ | 38.4 ± 12.2 | 39.3 ± 12.2 | 39.1 ± 12.2 | 0.40 |
| Diuretics | 95 (60.5) | 274 (65.2) | 369 (64.0) | 0.29 |
| ACE inhibitors or ARB | 71 (45.2) | 226 (53.8) | 297 (51.5) | 0.06 |
| Beta-blockers | 56 (35.7) | 164 (39.0) | 220 (38.1) | 0.45 |
| Statin | 86 (54.8) | 260 (61.9) | 346 (60.0) | 0.10 |
| **Echocardiographic data** |  |  |  |  |
| LVEF, % | 58.2 ± 15.5 | 57.3 ± 15.6 | 57.5 ± 15.6 | 0.50 |
| Mean transaortic gradient, mmHg | 45.8 ± 14.5 | 49.2 ± 16.6 | 48.2 ± 16.1 | **0.021** |
| AVA, cm^2^ | 0.69 ± 0.17 | 0.67 ± 0.19 | 0.67 ± 0.19 | 0.31 |
| **Procedural data** |  |  |  |  |
| Access site |  |  |  | 0.31 |
| Transfemoral approach | 144 (91.7) | 395 (94.0) | 539 (93.4) |  |
| Other | 13 (8.3) | 25 (6.0) | 38 (6.6) |  |
| Prosthesis type |  |  |  | **< 0.001** |
| Corevalve | 93 (59.2) | 333 (79.3) | 426 (73.8) |  |
| Sapien XT | 55 (35.0) | 78 (18.6) | 133 (23.1) |  |
| Inovare | 9 (5.7) | 9 (2.1) | 18 (3.1) |  |
| Contrast media volume, mL | 194 ± 115 | 181 ± 101 | 185 ± 105 | 0.26 |

Values are n (%) or mean (± SD).

Abbreviations: ACE, angiotensin-converting enzyme; AKI, acute kidney injury; ARB, angiotensin receptor blocker; AVA, aortic valve area; CABG, coronary artery bypass graft; CAD, coronary artery disease; COPD, chronic obstructive pulmonary disease; eGFR, estimated glomerular filtration rate; LVEF, left ventricular ejection fraction; NYHA, New York Heart Association; STS, Society of Thoracic Surgeons; TIRFI, TAVR induced renal function improvement.
