# Supplementary material for: Improvement of renal function after transcatheter aortic valve replacement in patients with chronic kidney disease
Source: PLoS One. 2021 May 13;16(5):e0251066. doi: 10.1371/journal.pone.0251066 (PMC8118303; doi:10.1371/journal.pone.0251066)
Supplement: S2 Table — (DOCX) [file pone.0251066.s002.docx]

**S2 Table. Predictors of TIRFI after TAVR procedure (including outcomes).**

| **Variable** | **Univariable Analysis** | | **Multivariable Analysis** | |
| --- | --- | --- | --- | --- |
|  | **OR (95% CI)** | **P value** | **OR (95% CI)** | **P value** |
| **Age, years** | 0.98 (0.95 – 1.00) | 0.13 | 0.97 (0.94 – 1.00) | **0.024** |
| **Male sex** | 0.84 (0.59 – 1.19) | 0.32 | **-** | **-** |
| **NYHA class III/IV** | 1.11 (0.69 – 1.76) | 0.67 | - | - |
| **Diabetes** | 0.76 (0.52 – 1.11) | 0.15 | 0.76 (0.51 – 1.13) | 0.18 |
| **Hypertension** | 1.09 (0.74 – 1.63) | 0.65 | - | - |
| **COPD** | 1.01 (0.65 – 1.56) | 0.98 | - | - |
| **Pulmonary hypertension** | 0.77 (0.51 – 1.16) | 0.20 | - | - |
| **CAD** | 0.73 (0.51 – 1.04) | **0.077** | 0.76 (0.52 – 1.10) | 0.14 |
| **Peripheral vascular disease** | 1.13 (0.72 – 1.78) | 0.60 | - | - |
| **Previous CABG** | 0.96 (0.62 – 1.46) | 0.83 | - | - |
| **STS score, %** | 1.00 (0.98 – 1.03) | 0.45 | - | - |
| **eGFR, mL/min/1.73m^2^** | 0.98 (0.96 – 0.99) | **0.014** | 0.97 (0.96 – 0.99) | **<0.001** |
| **Diuretics** | 1.15 (0.80 – 1.64) | 0.46 | - | - |
| **ACE inhibitors or ARB** | 1.27 (0.90 – 1.79) | 0.18 | 1.23 (0.86 – 1.77) | 0.26 |
| **Beta-blockers** | 1.06 (0.75 – 1.51) | 0.73 | - | - |
| **Statin** | 0.96 (0.68 – 1.37) | 0.83 | - | - |
| **LVEF, %** | 0.99 (0.98 – 1.00) | 0.45 | - | **-** |
| **Mean transaortic gradient, mmHg** | 1.00 (0.99 – 1.02) | 0.10 | 1.01 (1.00 – 1.02) | 0.057 |
| **AVA, cm^2^** | 1.68 (0.64 – 4.38) | 0.28 | - | **-** |
| **Contrast media volume, mL** | 1.00 (0.99 – 1.00) | 0.71 | - | **-** |
| **Procedure access (except Transfemoral)** | 0.58 ( 0.27 – 1.25) | 0.16 | 0.52 (0.23 – 1.14) | 0.10 |
| **Inovare prosthesis** | 0.54 (0.18 – 1.68) | 0.55 | - | **-** |
| **Myocardial infarction** | 0.96 (0.18 – 5.31 | >0.99 | - | **-** |
| **All stroke/TIA** | 1.04 (0.54 – 1.99) | 0.91 | - | **-** |
| **Major or life-threatening bleeding** | 0.84 (0.52 – 1.35) | 0.46 | - | **-** |
| **Major vascular complication** | 0.84 (0.48 – 1.47) | 0.54 | - | **-** |
| **New persistent LBBB** | 0.90 (0.61 – 1.33) | 0.61 | - | **-** |
| **Valve malpositioning** | 0.34 (0.12 – 0.99) | **0.039** | 0.25 (0.07 – 0.87) | **0.029** |
| **New pacemaker** | 0.87 (0.57 – 1.33) | 0.52 | - | **-** |

95% CI, 95% confidence interval.

Abbreviations: ACE indicates angiotensin-converting enzyme; ARB, angiotensin receptor blocker; AVA, aortic valve area; CABG, coronary artery bypass graft; CAD, coronary artery disease; COPD, chronic obstructive pulmonary disease; eGFR, estimated glomerular filtration rate; LBBB, left bundle branch block; LVEF, left ventricular ejection fraction; NYHA, New York Heart Association; OR, odds ratio; STS, Society of Thoracic Surgeons; TAVR, transcatheter aortic valve replacement; TIA, transient ischemic attack; TIRFI, TAVR induced renal function improvement.
