# Supplementary material for: Improvement of renal function after transcatheter aortic valve replacement in patients with chronic kidney disease
Source: PLoS One. 2021 May 13;16(5):e0251066. doi: 10.1371/journal.pone.0251066 (PMC8118303; doi:10.1371/journal.pone.0251066)
Supplement: S3 Table — (DOCX) [file pone.0251066.s003.docx]

**S3 Table. Predictors of AKI after TAVR procedure (including outcomes).**

| **Variable** | **Univariable Analysis** | | **Multivariable Analysis** | |
| --- | --- | --- | --- | --- |
|  | **OR (95% CI)** | **P value** | **OR (95% CI)** | **P value** |
| **Age, year** | 1.02 (0.99 – 1.05) | **0.06** | 1.03 (1.01 – 1.06) | **0.021** |
| **Male sex** | 0.83 (0.59 – 1.17) | 0.29 | **-** | **-** |
| **NYHA class III/IV** | 0.99 (0.63 – 1.56) | 0.95 | - | - |
| **Diabetes** | 0.17 (0.82 – 1.69) | 0.38 | - | - |
| **Hypertension** | 1.71 (0.13 – 2.59) | **0.011** | 1.72 (1.12 – 2.66) | **0.014** |
| **COPD** | 0.90 (0.58 – 1.40) | 0.65 | - | - |
| **Pulmonary hypertension** | 0.94 (0.63 – 1.40) | 0.75 | - | - |
| **CAD** | 0.99 (0.69 – 1.40) | 0.93 | - | **-** |
| **Peripheral vascular disease** | 1.07 (0.68 – 1.69) | 0.77 | - | - |
| **Previous CABG** | 0.74 (0.48 – 1.15) | 0.18 | 0.82 (0.52 – 1.30) | 0.40 |
| **STS score, %** | 1.00 (0.97 – 1.02) | 0.98 | - | - |
| **eGFR, mL/min/1.73m^2^** | 1.01 (0.99 – 1.02) | **0.008** | 1.01 (1.00 – 1.03) | 0.07 |
| **Diuretics** | 1.15 (0.80 – 1.64) | 0.44 | - | - |
| **ACE inhibitors or ARB** | 1.11 (0.79 – 1.57) | 0.54 | - | - |
| **Beta-blockers** | 0.93 (0.65 – 1.34) | 0.66 | - | - |
| **Statin** | 1.22 (0.86 – 1.74) | 0.26 | - | - |
| **LVEF, %** | 1.00 (0.99 – 1.01) | 0.28 | - | **-** |
| **Mean transaortic gradient, mmHg** | 1.00 (0.99 – 1.01) | 0.97 | - | **-** |
| **AVA, cm^2^** | 0.71 (0.26 – 1.87) | 0.49 | - | **-** |
| **Contrast media volume, mL** | 0.99 (0.99 – 1.00) | 0.60 | - | **-** |
| **Non-transfemoral access** | 0.93 (1.00 – 3.73) | **0.048** | 2.06 (1.03 – 4.13) | **0.042** |
| **Inovare prosthesis** | 1.46 (0.56 – 3.77) | 0.62 | - | **-** |
| **Myocardial infarction** | 1.86 (0.37 – 9.28) | 0.43 | - | **-** |
| **All stroke/TIA** | 1.51 ( 0.80 – 2.82) | 0.19 | 1.18 (0.61 – 2.30) | 0.625 |
| **Major or life-threatening bleeding** | 2.33 (1.49 – 3.64) | **<0.001** | 1.83 (1.08 – 3.08) | **0.024** |
| **Major vascular complication** | 1.93 (1.15 – 3.25) | **0.012** | 1.28 (0.70 – 2.34) | 0.43 |
| **New persistent LBBB** | 1.38 (0.95 – 2.01) | 0.09 | 1.24 (0.83 – 1.84) | 0.29 |
| **Valve malpositioning** | 3.71 (1.62 – 8.48) | **0.001** | 4.55 (1.90 – 10.9) | **0.001** |
| **New pacemaker** | 0.94 (0.62 – 1.43) | 0.78 | - | **-** |

95% CI, 95% confidence interval.

Abbreviations: ACE indicates angiotensin-converting enzyme; AKI, acute kidney injury; ARB, angiotensin receptor blocker; AVA, aortic valve area; CABG, coronary artery bypass graft; CAD, coronary artery disease; COPD, chronic obstructive pulmonary disease; eGFR, estimated glomerular filtration rate; LBBB, left bundle branch block; LVEF, left ventricular ejection fraction; NYHA, New York Heart Association; OR, odds ratio; STS, Society of Thoracic Surgeons; TAVR, transcatheter aortic valve replacement; TIA, transient ischemic attack; TAVR, transcatheter aortic valve replacement.
